# Synthesis of Gelatin Methacryloyl Analogs and Their Use in the Fabrication of pH-Responsive Microspheres

**DOI:** 10.3390/pharmaceutics16081016

**Published:** 2024-07-31

**Authors:** Karolina Valente, Geneviève N. Boice, Cameron Polglase, Roman G. Belli, Elaina Bourque, Afzal Suleman, Alexandre Brolo

**Affiliations:** 1 VoxCell BioInnovation Inc., Victoria, BC V8T 5L2, Canada; kvalente@voxcellbio.com (K.V.); gboice@voxcellbio.com (G.N.B.); cpolglase@voxcellbio.com (C.P.); 2 Department of Chemistry, University of Victoria, Victoria, BC V8P 5C2, Canada; rbelli@uvic.ca (R.G.B.); elaina.bourque@gmail.com (E.B.); 3 Department of Mechanical Engineering, University of Victoria, Victoria, BC V8P 5C2, Canada; suleman@uvic.ca; 4 Centre for Advanced Materials and Related Technology, University of Victoria, Victoria, BC V8P 5C2, Canada

**Keywords:** pH-responsive hydrogel, gelatin methacryloyl, hydrogel microspheres, flow-focusing microfluidic device, drug delivery systems

## Abstract

pH-responsive hydrogels have numerous applications in tissue engineering, drug delivery systems, and diagnostics. Gelatin methacryloyl (GelMA) is a biocompatible, semi-synthetic polymer prepared from gelatin. When combined with aqueous solvents, GelMA forms hydrogels that have extensive applications in biomedical engineering. GelMA can be produced with different degrees of methacryloyl substitution; however, the synthesis of this polymer has not been tuned towards producing selectively modified materials for single-component pH-responsive hydrogels. In this work, we have explored two different synthetic routes targeting different gelatin functional groups (amine, hydroxyl, and/or carboxyl) to produce two GelMA analogs: gelatin A methacryloyl glycerylester (polymer A) and gelatin B methacrylamide (polymer B). Polymers A and B were used to fabricate pH-responsive hydrogel microspheres in a flow-focusing microfluidic device. At neutral pH, polymer A and B microspheres displayed an average diameter of ~40 µm. At pH 6, microspheres from polymer A showed a swelling ratio of 159.1 ± 11.5%, while at pH 10, a 288.6 ± 11.6% swelling ratio was recorded for polymer B particles.

## 1. Introduction

Hydrogels are three-dimensional networks composed of polymeric materials (natural or synthetic) that can absorb a considerable amount of water and swell in aqueous medium, while maintaining physical integrity. These characteristic hydrogel properties are conferred by the presence of hydrophilic functional groups (e.g., -OH, -COOH, -SO_3_H, -NH_2_, -CONH_3_) in the polymers [1]. Hydrogels have been extensively applied in the field of tissue engineering [2,3] and as drug delivery systems [4,5] due to their biocompatibility and resemblance to natural tissue.

Depending on the functional groups present on the polymeric chains, some hydrogels can respond to specific environmental changes such as temperature, light, and pH [6]. In the case of pH-responsive hydrogels, polymers containing acidic and basic pendants can accept or release protons according to the pH of the solution [7]. For hydrogels containing carboxyl groups, they release protons and become negatively charged at pH values higher than the pKa of -COOH/-COO- pair, forming anionic polyelectrolytes. Hydrogels containing amine groups, on the other hand, become positively charged at pH values lower than the pKa of -NH_3_+/-NH_2_ conjugate pair. The change in functional group charge in pH-responsive hydrogels under acidic or basic conditions leads to enhanced repulsion or attraction within the polymer strands and affects the water absorbing capabilities of the hydrogel, which causes pH-induced swelling and/or shrinking of the hydrogel. This ability has led to pH-responsive hydrogels being explored for numerous applications, ranging from controlled drug carriers and delivery systems to sensors [8,9].

The use of pH-responsive hydrogels for targeted drug delivery has been investigated as a way to overcome a variety of disease processes and physiological conditions that can complicate or prevent drugs from reaching the desired site of therapeutic action [6,10]. In oncology, the pH of the tumor environment can make drug delivery challenging. Cancer cells within a healthy tissue remodel the tissue environment, resulting in stiffening of the extracellular matrix and a concomitant increase in interstitial pressure [11]. The latter, combined with the disorganized structure of the tumor area, affects the clearance of waste products [12], leading to a decrease in pH in the tumor compared to surrounding tissue. The difference between the pH in the tumor (as low as 5.6 [13]) and the pH of the surrounding tissue (often ~7) can mean that drugs that are able to diffuse effectively in the healthy tissue cannot permeate the tumor environment. By encapsulation of drugs in pH-responsive cationic hydrogels, passage of the drug to the tumor is possible, and drug release within the tumor is enabled by the swelling of the hydrogel in the acidic environment, allowing release of the drug from the hydrogel [14,15]. Similarly, anionic hydrogels have been applied as oral drug delivery systems, since they can passage drugs through acidic conditions in the stomach to deliver drugs to the higher pH environment of the small intestine [16,17].

Gelatin is a natural polymer derived from collagen through a denaturation process. Depending on the denaturation process (acidic or basic treatment), either gelatin type A or gelatin type B are obtained [18]. Gelatin type A and B are similar, but differ in relative amounts of amino acid residues and isoelectric point. Gelatin has been vastly used in biomedical applications due to its ease of use, biocompatibility, and low cost compared to other polymers. Gelatin methacryloyl is a semi-synthetic biomaterial prepared from the incorporation of methacrylate and methacrylamide groups in gelatin via reaction with methacrylic anhydride [19,20] or glycidyl methacrylate [21]. The presence of arginine-glycine-aspartic acid (RGD) sequences on the gelatin backbone of GelMA promotes cell attachment and results in a biomaterial suitable for a variety of biomedical applications [22,23]. While most GelMA synthesis protocols focus on the reaction between methacrylate groups and amino residues, resulting in a decrease in free lysine groups [20,24], the reaction of methacrylate groups with hydroxyl and carboxyl groups in the gelatin backbone has also been reported to occur under certain conditions [25]. GelMA has been investigated as a copolymer in multi-component pH-responsive mixtures (e.g., PVDT-GelMA, GelMA/HA-CHO, and PAA-co-GelMA) [26,27,28], but to our knowledge, it has not been investigated as a single-component pH-responsive material. In this work, we investigate the effect of functional group substitution on the pH-dependent properties of single-component GelMA-based hydrogels.

We report the synthesis of two selectively methacrylated GelMA analogs, polymers A and B (synthesis performed by VoxCell BioInnovation), which demonstrate pH-dependent swelling behavior. The syntheses were tuned to favor selective modification of either carboxyl/hydroxyl or amino groups on the gelatin backbone [23,29]. In the case of polymer A, the synthesis was modified to maintain available amine groups while reacting hydroxyl and carboxyl groups with glycidyl methacrylate. In the case of polymer B, carboxyl and hydroxyl groups remained unreacted, while amine groups were allowed to react with methacrylic anhydride. Microspheres of polymers A and B were produced using a flow-focusing microfluidic device and mineral oil/span 80 as the continuous phase. The swelling and shrinking behavior of the GelMA-based microspheres in solutions of different pH values was determined by quantifying the degree of pH-dependent volume changes.

## 2. Materials and Methods

### 2.1. Materials

Gelatin from porcine skin (Type A, gel strength 300), gelatin from bovine skin (Type B), methacrylic anhydride (MAA) (contained 2000 ppm topanol A as inhibitor, 94%), glycidyl methacrylate (GMA) (≥97%), 2-hydroxy-4′-(2-hydroxyethoxy)-2-methylpropiophenone (Irgacure 2959, 98%), Span 80 (nonionic surfactant), mineral oil (light), and phosphate-buffered saline (PBS, pH 7.4) were purchased from Sigma-Aldrich. Tetrahydrofuran (THF, 99.9%), hydrochloric acid (36.5–38.0%), and dialysis membrane tubing (12,000 to 14,000 Da MWCO) were purchased from Fisher Scientific. Fluorescent carboxylated PS latex particles (CAF-100 nm) were purchased from Magsphere Inc. (Pasadena, CA, USA) SU-8 and SU-8 developer were purchased from Kayaku Advanced Materials. Sylgard 184 Silicone Elastomer Kit was purchased from Dow Corning. Silicon wafers (76.2 mm, P-type, Boron) were purchased from Silicon Materials Inc. (Glenshaw, PA, USA).

### 2.2. Synthesis of Polymers A and B

**Figure 1:**
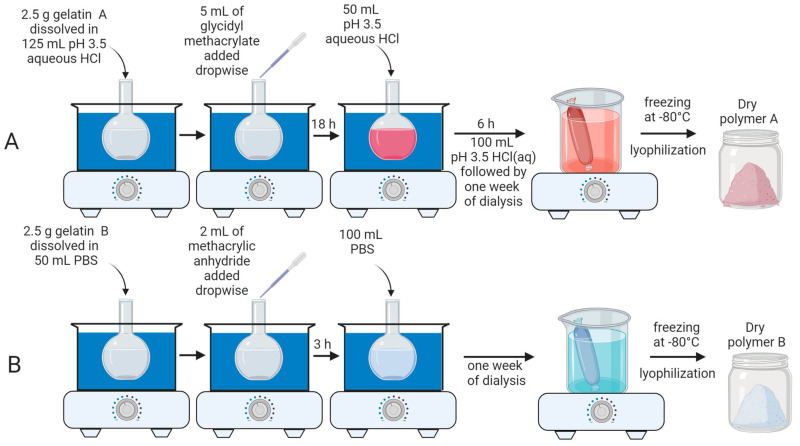
Synthesis of polymers A and B. (**A**) Polymer A was produced by reacting gelatin A with glycidyl methacrylate at pH 3.5. After a total reaction time of 24 h, 100 mL of pH 3.5 HCl solution was added to the aqueous mixture. The final solution was then dialyzed, followed by lyophilization to obtain polymer A. (**B**) Polymer B was produced by reacting gelatin B with methacrylic anhydride. After a total reaction time of 3 h, 100 mL of PBS was added to the solution, followed by dialysis and lyophilization.

Polymer A was synthesized from gelatin type A and glycidyl methacrylate (GMA). As illustrated by Figure 1A, the reaction was performed in a water bath at 40 °C. Initially, 2.5 g of gelatin type A was dissolved in 125 mL of aqueous solution of HCl (pH 3.5) in a round-bottom flask while stirring at 400 rpm. After all gelatin A was dissolved, 5 mL of glycidyl methacrylate was added dropwise (rate 0.5 mL/min) to the solution. The pH of the mixture was adjusted back to 3.5 every 30 min using HCl throughout the reaction. After the reaction had run for 18 h, 50 mL of pH 3.5 HCl solution was added to the mixture, and the reaction continued for an additional 6 h. After the total reaction time of 24 h, 100 mL of HCl solution was added to the round-bottom flask and the mixture was stirred for 10 min. The solution was then transferred to dialysis membranes and dialyzed with water at 40 °C for one week to eliminate unreacted glycidyl methacrylate. The solution was then frozen at −80 °C and lyophilized to obtain the dry product, polymer A.

Polymer B was synthesized by the reaction of gelatin type B with methacrylic anhydride (MAA). As shown in Figure 1B, 2.5 g of gelatin B was dissolved in 50 mL of PBS in a round-bottom flask while stirring at 400 rpm. Once all gelatin B was dissolved, 2.0 mL of methacrylic anhydride was added dropwise (rate 0.5 mL/min) to the solution. The mixture was allowed to react for 3 h, and the temperature was kept constant at 40 °C by a water bath. After 3 h, 100 mL of PBS was added to the reaction mixture, followed by dialysis against water at 40 °C for one week, to eliminate unreacted methacrylic anhydride. The dry polymer B was isolated following freezing at −80 °C and lyophilization.

### 2.3. Characterization of Polymers A and B

^1^H NMR measurements were recorded on a Bruker Avance 500 MHz spectrometer. Polymer samples were dissolved in 1 mL of deuterium oxide (D2O) containing a known amount of 3-(trimethylsilyl)propionic-2,2,3,3,-d4 acid sodium salt (TMSP) as an internal standard (δ (^1^H) = 0 ppm). The amount of methacryl groups (AM) in the samples was calculated as described previously [30]. Equation (1) was used to calculate the amount (in mmol per g of polymer sample) of methacryl groups (AM), while Equation (2) was used to calculate the degree of methacrylation of lysine groups (DMlysine) [31].

(1) $$AM\left(mmolg^{-1}\right)=\frac{\int methacryl\;peaks}{\int TMSP}\times \frac{9H}{1H}\times \frac{mmol(TMSP)}{g(polymer\;sample)}$$

(2) $${DM}_{lysine}=\left(1-\frac{\int lysine\;in\;polymer\;sample}{\int lysine\;in\;gelatin}\right)\times 100\%$$

### 2.4. Fabrication of Microfluidic Device

A flow-focusing microfluidic device was fabricated using standard photolithography. Briefly, SU-8 was spin coated on the surface of a silicon wafer to a final thickness of 150 μm. The wafer was soft baked at 65 °C for 30 min and then at 95 °C for 60 min. Using a photolithography mask, the flow-focusing design was patterned on the SU-8 layer by exposing it to UV light. The patterned wafer was post-baked at 95 °C for 90 min, followed by development using SU-8 developer. Polydimethylsiloxane (PDMS) was prepared by mixing a 10:1 (*w*/*w*) silicone elastomer base and curing agent. PDMS was then deposited at the surface of the SU-8 patterned silicon wafer. After degassing in a vacuum chamber, the assembly was baked at 100 °C for 3 h, to allow PDMS polymerization and hardening. After 3 h, the PDMS was peeled off from the wafer and holes were punched in the inlets and outlets of the channels. The PDMS layer was permanently bonded to a glass slide using plasma cleaning. The resulting chips were then baked at 60 °C for at least 3 h to allow hydrophobicity to be restored in the PDMS channels. The design of the microfluidic device consisted of an inner channel for the dispersed phase and two outer channels for the continuous phase.

### 2.5. Synthesis and Characterization of Hydrogel Microspheres

Hydrogel microspheres were produced in a flow-focusing microfluidic device. A 5% (*w*/*v*) solution of polymer A or B was prepared using PBS containing 0.5% (*w*/*v*) Irgacure 2959. This solution was used as the dispersed phase inside the microfluidic device. The continuous phase was composed of a 4:1 (*w*/*w*) ratio of mineral oil (16.8 g):Span 80 (4.2 g). Flow rates of 1 μL/min and 20 μL/min were used for the dispersed and continuous phases, respectively. The outlet tubing was cut to two meters total length and positioned in a 3D printed support, creating a meander. A 365 nm UV light was placed on top of the meander, allowing microspheres leaving the microfluidic device to be exposed to UV light for 10 min before being collected. After collection, the microspheres were allowed to stabilize for 12 h in the dark at room temperature. A volume of 0.5 mL of suspension containing the microspheres was rinsed using 1 mL of THF, followed by centrifugation for 7 min at 8000 rpm. This washing procedure was repeated three times. The microspheres were then stored in 0.5 mL of PBS prior to use, with a final concentration of 2 × 10^6^ microspheres/mL. For the generation of fluorescently labeled microspheres, 20 µL of fluorescent polystyrene nanoparticles were added to a 0.5 mL solution of polymer A or B, and the hydrogel microspheres preparation proceeded as described above.

The impact of pH on the diameter of the microspheres was investigated by incubating 200 µL of particle suspensions in 1 mL of aqueous solutions with pH of 6, 7.4, and 10 for 2 h. The change in diameter was observed using the bright field camera on a laser scanning microscope (Zeiss LSM 880). In the case of the microspheres containing polystyrene nanoparticles, fluorescent images were captured with the LSM. The excitation was observed at 543 nm (helium-neon laser, 40% laser power), and the emission band was 560–700 nm. Images were collected at a frame size of 0.35 µm × 0.35 µm (4096 pixels × 4096 pixels), a pixel time of 1.03 µs, and a frame time of 162.32 s using an EC Plan-Neofluar 10×/0.30 M27 objective lens.

The diameters of 100 microspheres were measured for each experiment using the measure tool on Zen 2.3 software in both bright field and fluorescent images. The particle diameter was obtained by averaging the measurement for each experiment (100 microspheres) and calculating the standard deviation. The experiments were repeated three times from different batches of polymers A and B. The swelling ratio was calculated for each experiment based on the fractional increase in particle volume as reported elsewhere [32], using Equations (3) and (4) for swelling and shrinkage percentages for polymers A and B. The reported swelling and deswelling ratios are the averages of three experiments, with corresponding standard deviations.

(3) $$Q_{pH6\;or\;10-PBS}=\left(\frac{D_{pH6}^{3}-D_{PBS}^{3}}{D_{PBS}^{3}}\right)\times 100\%$$

(4) $$Q_{pH10\;or\;6-PBS}=\left(\frac{D_{pH10}^{3}-D_{PBS}^{3}}{D_{PBS}^{3}}\right)\times 100\%$$

where D_pH6_, D_pH10_, and D_PBS_ correspond to the diameters of the particles in pH 6, pH 10, and PBS, respectively.

## 3. Results and Discussion

### 3.1. Determination of Degree of Modification of Polymers A and B

**Figure 2:**
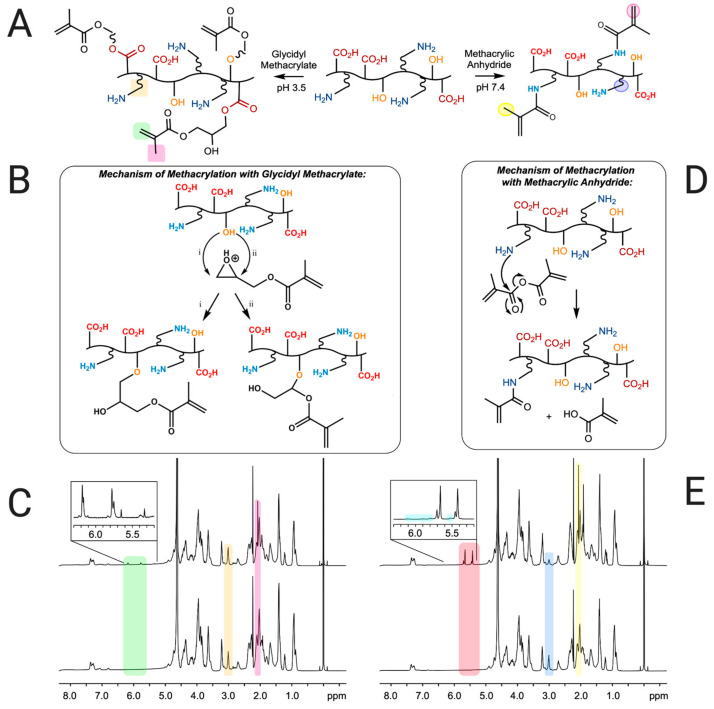
Synthesis of polymers A and B. (**A**) Two synthesis routes from gelatin. The reaction of gelatin A with glycidyl methacrylate at pH 3.5 produced polymer A (left), while the reaction of gelatin B with methacrylic anhydride at pH 7.4 generated polymer B (right). (**B**) Mechanism of methacrylation of gelatin type A hydroxyl groups with glycidyl methacrylate through ring-opening reaction. (**C**) ^1^H NMR spectra of polymer A (top) and gelatin type A (bottom). (**D**) Mechanism of methacrylation of gelatin type B with methacrylic anhydride. (**E**) ^1^H NMR spectra of polymer B (top) and gelatin type B (bottom).

Polymer A was synthesized by reacting gelatin type A with glycidyl methacrylate at pH 3.5 (see Figure 1A, experimental section and Figure 2A for a detailed description of the synthesis). Under acidic conditions, glycidyl methacrylate has been shown to react with hydroxyl and carboxylic acid groups of polyvinyl alcohol and polyacrylic acid polymers, yielding products consistent with epoxide ring-opening mechanisms [33]. In the case of the hydroxyl groups in the gelatin A backbone, this reaction likely proceeds through direct nucleophilic attack of the hydroxyl on the protonated epoxide, leading to the formation of 3-methacryloyl-1-glycerylester (GMA1) and 3-methacryloyl-2-glycerylester (GMA2) substituents (Figure 2B). In the case of carboxylic acid groups in gelatin, the reaction may occur stepwise through the formation of 3-methacryloyl-1-glycerol and 3-methacryloyl-2-glycerol via epoxide ring opening by water, followed by acid-catalyzed esterification at the carboxylic acid to form GMA1 and GMA2 moieties. The presence of GMA1 and GMA2 substituents in polymer A was verified by ^1^H NMR, presented in Figure 2C. The overlapping ^1^H NMR signals observed at 6.18 and also at 5.76 ppm (indicated in the green region in Figure 2C) correspond to the geminal vinyl hydrogens of both GMA1 and GMA2. The chemical shift difference induced by a hydroxyl versus a carboxyl attachment is small and cannot be distinguished at the NMR field strength used in this study, leading to overlapping signals for these species in the spectrum. The additional peak at 2.07 ppm seen in Figure 2C (pink region) is associated to the methyl carbon-linked hydrogens at the vinyl carbon, which also confirms the modification of the polymer backbone by GMA.

The extent of chemical modification, determined using Equation (1), was 0.103 mmol of methacryl groups/g of polymer A. The integration of the peak at 6.18 ppm (green region in Figure 2C) was used to provide the total amount of methacryl groups, including GMA1 and GMA2, in polymer A.

Additional peaks were also seen within the green region of Figure 2C (see inset) at 5.33 and 5.65 ppm. These peaks could be an indication of side reactions involving GMA and amine groups in the polymer backbone. Amine-epoxy reactions are expected to display ^1^H NMR signatures correspondent to the vinyl groups at 6.2–5.8, but vinyl peaks from amine-methacryoyl reactions are consistent with the observed chemical shifts. While the DMlysine values calculated from the integration of lysine peaks at 3.00 ppm (Equation (2), yellow region in Figure 2C) were around 0%, amine protons are exchangeable with deuterium under the NMR conditions, and DMlysine values derived from integration may not accurately reflect the presence of small amounts of lysine substitution. Under acidic conditions, the lysine amine groups are considered to be fully protonated, and are therefore unlikely to act as nucleophiles; however, it is possible that fluctuations in pH towards the isoelectric point of gelatin during the reaction could have resulted in the formation of small amounts of methacrylamide.

The synthesis of polymer B involved the reaction of gelatin type B with methacrylic anhydride at pH 7.4, as indicated in Figure 2A (see Figure 1B and the experimental section for a detailed description of the synthesis). The conditions of the synthesis involved a higher pH value than the isoelectric point of gelatin type B (between 5 and 6) to favor a higher degree of methacrylation [25]. Methacrylic acid is a by-product as the reaction proceeds, which lowers the pH of the solution during the synthesis. Previous reports have indicated that readjusting the pH during reaction of gelatin A with methacrylic anhydride loadings similar to those used here results in a higher degree of lysine substitution [25], and can be accompanied by increased reaction at hydroxyl/carboxyl groups [29]. Because we desired to selectively substitute lysine, we performed the synthesis of polymer B (Figure 2D) without pH readjustment. Figure 2E shows ^1^H NMR data that confirms the formation of the desired product. The decrease in the lysine peak at 3.00 ppm (blue region in Figure 2E) indicates the functionalization of free amine groups of the gelatin B. In addition, Figure 2E displays prominent peaks at 5.65 and 5.42 ppm (pink region), which are assigned to the geminal protons of the methacrylamide groups’ vinyl moiety. Additionally, the peak at 2.07 ppm in the ^1^H NMR spectrum of polymer B (yellow region in Figure 2E) can be assigned to the methyl hydrogens of the methacryl group, confirming the reaction with MAA. The very small peak at 6.11 ppm suggests the presence of a small amount of methacrylate groups arising from MAA reaction with hydroxyl groups, although the other methacrylate vinyl proton is overlapped with the methacrylamide peak at 5.65 ppm (Figure 2E inset). The integration of the peak at 5.65 ppm represents, then, the total amount of methacryl groups present in polymer B.

The DMlysine value, obtained from the integration of the lysine peak at 3.00 ppm, was 58%. This relatively low DM value is due to the formation of methacrylic acid during the synthesis, which lowers the pH, resulting in a lower DS value [25]. Values of 0.227 mmol of methacrylamide groups/g for polymer B and 0.062 mmol of methacrylate groups/g for polymer B were obtained (Equation (1)) from the integration of the peaks at 5.65 ppm (methacrylamide and methacrylate) and at 6.11 ppm, respectively.

### 3.2. Fabrication of Hydrogel Microspheres

**Figure 3:**
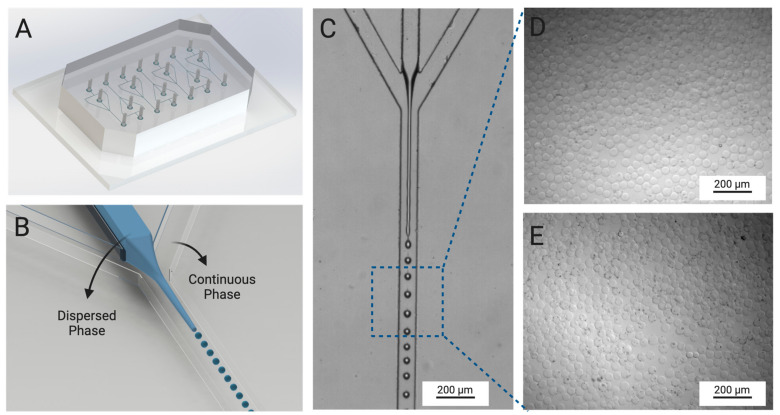
Fabrication of GelMA microspheres. (**A**) Microspheres were fabricated inside of a flow-focusing microfluidic device. (**B**) The device contained two phases (continuous and dispersed). The dispersed phase was composed of 5% (*w*/*v*) GelMA (polymer A or B), while the continuous phase consisted of mineral oil with Span 80. (**C**) Flow profile during production of microspheres. (**D**) Optical microscopy (OM) image of polymer A microspheres. (**E**) OM image of polymer B microspheres.

A flow-focusing microfluidic device, presented in Figure 3A, was used for the fabrication of hydrogel microspheres. In the inner channel of the microfluidic device, a solution containing 5% (*w*/*v*) polymer solution (either polymer A or polymer B) and 0.5% (*w*/*v*) Irgacure 2959 (photoinitiator) was set to flow at a rate of 1 µL/min. GelMA-based solutions are highly viscous at room temperature; therefore, a temperature controlled chamber was created around the syringe pump and tubing to maintain the solution temperature at 40 °C during the flow. A mineral oil/Span 80 mixture was injected in the outer channels of the device (Figure 3B). The surfactant (Span 80) was added to the mineral oil to preclude aggregation of the microspheres inside the microfluidic device. To develop a stream of dispersed phase, a flow-focusing region was created at the cross-junction of the device where the immiscible polymer and mineral oil phases intersect. Shear forces and perturbation in the flow [34,35,36] then created spherical particles (microspheres) of the dispersed phase (Figure 3C).

The GelMA microspheres leaving the outlet of the microfluidic device entered a 2 m meander tubing region that was exposed to UV light. The GelMA particles were exposed to UV light in the meander for 10 min, activating the radical photoinitiator Irgacure 2959 and transforming the GelMA particles into crosslinked hydrogel microspheres. The hydrogel microspheres were then collected, stored, and allowed to stabilize overnight in the dark. The hydrogel microspheres were washed in THF to remove the oil/surfactant mixture and the excess photoinitiator, placed in PBS solution for 24 h, and then used within 3 days. Figure 3D,E show that the microspheres were uniform in size and shape, with diameters of 39.35 ± 2.59 μm and 38.63 ± 2.30 μm for particles produced from polymers A and B, respectively.

### 3.3. Swelling and Shrinking Behavior of Hydrogel Microspheres

**Figure 4:**
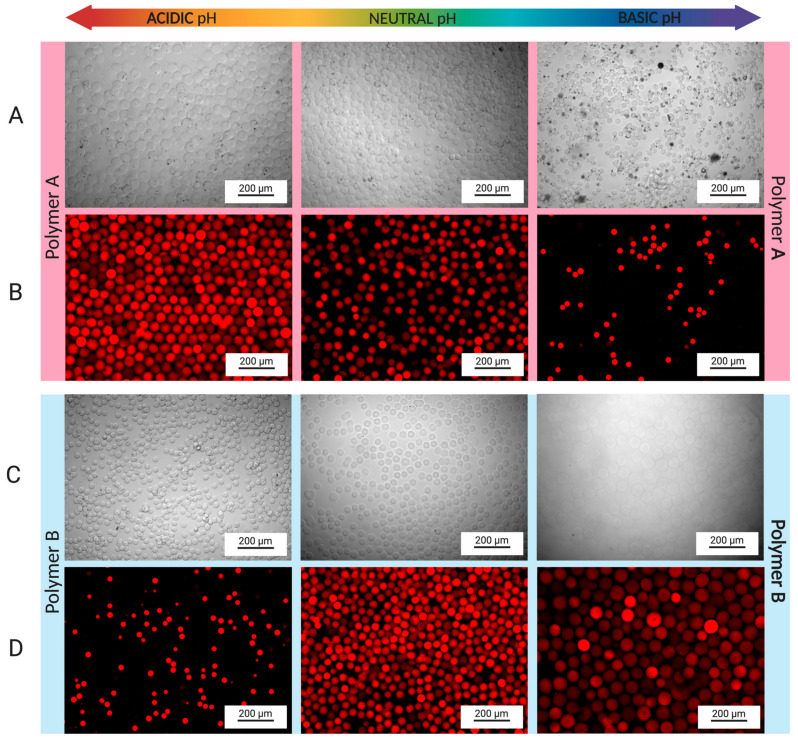
Swelling behavior of GelMA microspheres. (**A**) Polymer A microspheres displayed swelling at acidic pH (6.0), while shrinkage was seen at basic pH (10). (**B**) Swelling and shrinking of polymer A microspheres was also investigated by adding red-fluorescent PS nanoparticles to the hydrogel solution. The same swelling and de-swelling behaviors were observed for the fluorescent microspheres. (**C**) The opposite behavior was observed for polymer B microspheres, in which swelling was observed at basic pH, while shrinkage was seen in an acidic environment. (**D**) Microspheres were also fabricated by adding PS particles to polymer B pre-hydrogel solution, providing easier observation of the increase and decrease in the diameter of the microspheres.

**Figure 5:**
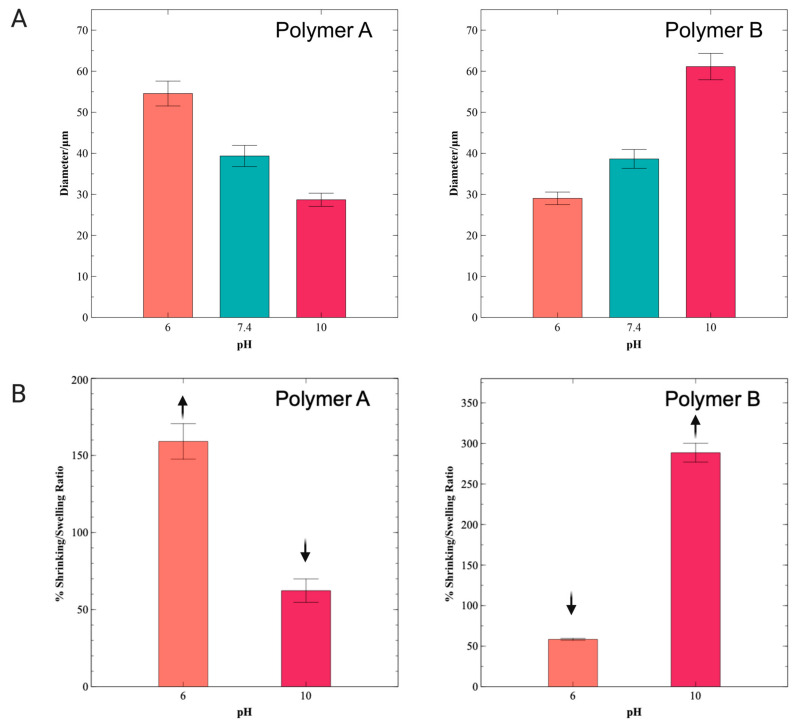
Swelling and de-swelling behavior of GelMA microspheres. (**A**) Change in diameter was observed for polymers A and B, according to the pH of the environment. For polymer A, a decrease in diameter was seen with an increase in pH, while the opposite behavior was observed for polymer B (increase in diameter with increase in pH). (**B**) Swelling ratios of 159.1 ± 11.5% and 288.6 ± 11.6% were obtained for polymers A and B, respectively, at pH 6 and pH 10. Shrinking ratios of 62.3 ± 7.6% and 58.4 ± 1.2% were observed for polymer A at pH 10 and for polymer B at pH 6, respectively.

Hydrogels swell due to expansion of their hydrophilic chains when in contact with an aqueous environment. Ionizable functional groups in the polymer chain can either accept or release protons in response to the pH of the environment. Polymer A synthesis was conducted at pH 3.5, at which pH most gelatin amine groups should be protonated, and therefore, unavailable to react with glycidyl methacrylate. Consequently, the hydroxyl and carboxyl groups of polymer A were modified by substitution with GMA1 and GMA2, while the amine groups remained largely unreacted. Figure 4A shows bright field microscopy images displaying the response of the polymer A hydrogel microspheres upon exposure to solutions of different pH values. Polymer A microspheres increase in diameter in acidic solutions and decrease in diameter in basic solutions. The swelling in acidic solutions can be explained by the protonation of amine groups, which induces both intra- and interchain repulsions and favors polymer hydration, eventually increasing the overall volume of the materials (Figure 4A). Swelling and de-swelling were also investigated by encapsulating red fluorescent PS nanoparticles in the polymer A solution prior to the production of the microspheres (Figure 4B). Figure 4B displays a more obvious change in the particle diameter, allowing for better visualization and quantification. De-swelling (shrinking) of polymer A microspheres was observed at pH 10. Polymer A microspheres displayed diameters of 39.4 ± 2.6 μm in a pH 7.4 environment, 54.6 ± 3.0 μm in a pH 6 environment, and 28.7 ± 1.6 μm in a pH 10 environment. Figure 5A summarizes the diameters of the hydrogel microspheres measured from the fluorescent images in Figure 4. Figure 5B shows plots of particle volume variation (swelling and de-swelling ratios; Equation (3)) measured with respect to the particle volume at pH = 7.4 as reference (100% value). The microspheres generated from polymer A displayed a swelling ratio of 159.1 ± 11.5% (Equation (3)) at pH 6 and a shrinking ratio (Equation (4)) of 62.3 ± 7.6% at pH 10 (Figure 5B).

In contrast to polymer A microspheres, the swelling behavior of polymer B hydrogel microspheres is driven by the effect of unreacted carboxyl and hydroxyl groups. Although the DMlysine of 58% implies that a substantial amount of amine remained unmodified at the polymer backbone, amino acid composition analysis has shown that gelatin B contains fewer lysine residues (per 1000 total amino acid residues) than gelatin A [37]. In addition, gelatin type B contains more free carboxyl groups per 100 g of gelatin than gelatin type A [38]. Thus, in basic conditions, polymer B hydrogel microspheres displayed swelling (Figure 4C) due to the ionization of carboxyl side chain groups, which results in both ionic repulsion within the polymer and enhanced attractive interactions with water molecules that surround the polypeptide chains. A decrease in microsphere size was observed at pH 6. The pH-induced change in the diameter of the polymer B microspheres was investigated using fluorescent PS particles introduced in the polymer B pre-hydrogel solution (Figure 4D). These fluorescently tagged microspheres exhibited diameters of 38.6 ± 2.3 μm in a pH 7.4 environment, 29.0 ± 1.5 μm in a pH 6 environment, and 61.1 ± 3.2 μm in a pH 10 environment (Figure 5A). Swelling of 288.6 ± 11.6% was observed at pH 10, while 58.4 ± 1.2% de-swelling was observed at pH 6 (Figure 5B). For both polymer A and B microspheres, swelling and de-swelling processes were fast (<2 h) and were shown to be reversible.

## 4. Conclusions

Two synthetic methods for producing pH-responsive gelatin methacryloyl polymers were reported. The two methods prioritized the selective methacrylation of either gelatin hydroxyl/carboxyl groups or gelatin amine groups. The resulting polymers were rich in either free amine groups (polymer A) or free carboxyl/hydroxyl groups (polymer B), as characterized by ^1^H NMR. Microspheres of polymer A and polymer B demonstrated pH-dependent swelling and de-swelling behavior. Polymer A microspheres showed swelling at pH 6 and shrinking at pH 10, while polymer B microspheres displayed shrinking at pH 6 and swelling at pH 10. The contrasting swelling trends observed for polymers A and B were attributed to the ability of amines to form cations via protonation under acidic conditions, leading to swelling at low pH (polymer A), and to carboxyl groups’ ability to form anions via deprotonation under basic conditions, leading to swelling at high pH (polymer B). In the case of the microspheres produced from polymer B, even though some free amine groups were present, as characterized by NMR, it is presumed that the relative overabundance of unreacted carboxyl groups resulted in an anionic hydrogel displaying swelling at high pH values. Swelling and shrinking ratios of 159.1 ± 11.5% and 62.3 ± 7.6%, respectively, were obtained for polymer A. In the case of polymer B, ratios of 288.6 ± 11.6% and 58.4 ± 1.2% were recorded for swelling and shrinking, respectively.

GelMA has been shown to be an excellent biomaterial for tissue engineering [39,40], 3D printing [41,42] and drug delivery systems [43,44]. The presence of RGD sequences in its gelatin backbone enhances cell attachment and biocompatibility. Although the diversity of functional groups on the gelatin backbone is well known, to our knowledge, selective modification of these functional groups towards the development of single-component pH-responsive hydrogels has not yet been explored. The pH-dependent swelling/de-swelling behavior of the GelMA-based microspheres described in this study amplifies the potential use of this biomaterial as hydrogels in pH-responsive applications, ranging from drug delivery systems to sensors.

## 5. Patent

Valente, Karolina et al. VoxCell BioInnovation, Inc. pH Responsive Polymers and Related Methods of Synthesizing, Fabricating, and Deploying pH Responsive Polymers. International Publication Number WO 2023/155002 A1. 24 August 2023.

## Data Availability

Data are contained within the article.
